# Fluoroquinolone structure and translocation flux across bacterial membrane

**DOI:** 10.1038/s41598-017-08775-4

**Published:** 2017-08-29

**Authors:** Julia Vergalli, Estelle Dumont, Bertrand Cinquin, Laure Maigre, Jelena Pajovic, Eric Bacqué, Michael Mourez, Matthieu Réfrégiers, Jean-Marie Pagès

**Affiliations:** 1 UMR_MD1, Aix-Marseille Univ, IRBA, TMCD2 Marseille, France; 2 DISCO beamline, Synchrotron Soleil, Saint-Aubin, France; 3 University of Belgrade, Faculty of Physics, 11001 Belgrade, Serbia; 4 Infectious Diseases Therapeutic Area, Sanofi R&D, Marcy l’Etoile, France; 5 Present Address: LBPA, ENS CACHAN, Cachan, France

**Keywords:** Antimicrobial resistance, Bacterial infection, Porins

## Abstract

Bacterial multidrug resistance is a worrying health issue. In Gram-negative antibacterial research, the challenge is to define the antibiotic permeation across the membranes. Passing through the membrane barrier to reach the inhibitory concentration inside the bacterium is a pivotal step for antibacterial molecules. A spectrofluorimetric methodology has been developed to detect fluoroquinolones in bacterial population and inside individual Gram-negative bacterial cells. In this work, we studied the antibiotic accumulation in cells expressing various levels of efflux pumps. The assays allow us to determine the intracellular concentration of the fluoroquinolones to study the relationships between the level of efflux activity and the antibiotic accumulation, and finally to evaluate the impact of fluoroquinolone structures in this process. This represents the first protocol to identify some structural parameters involved in antibiotic translocation and accumulation, and to illustrate the recently proposed “Structure Intracellular Concentration Activity Relationship” (SICAR) concept.

## Introduction

A main challenge in antibacterial chemotherapy is to determine and carefully use the *in situ* parameters that modulate the activity of drugs in order to improve the efficacy of the antibacterial molecules (see the last WHO report, http://www.who.int/antimicrobial-resistance/en/). This is particularly important with the continuing emergence and the spreading of multidrug resistant bacteria (MDR) that contribute to therapeutic failure^1–4^. Several papers illustrate the intensity and relevance of the concern in clinical isolates and ask for a better understanding of membrane permeation and intracellular concentration of antibiotics, which is urgently required to combat MDR pathogens. Indeed, passing the membrane barrier to reach a threshold concentration inside the bacterial cell is a pivotal step in antibacterial action^5–9^. This key point has not been strongly addressed until now or only in few specific cases with characterized bacterial strains^8–11^.

As recently reported^8, 12–15^, there is a large collection of methods available to measure efflux activity, but their sensitivity and validity must be clearly demonstrated in the different conditions used. Thus, the lack of an appropriate well-defined assay is a serious bottleneck for the optimization of an antibiotic intra-bacterial accumulation, the first inescapable step for understanding antimicrobial activity. Recently, the intracellular accumulation of a fluoroquinolone has been studied with a synchrotron light source using intrinsic bacterial fluorescence as a powerful internal standard for correcting biological variations associated with bacterial samples^16^. In this case, the fluoroquinolone antibiotic fleroxacin exhibits sufficient changes in fluorescence intensity to monitor its accumulation in single bacterial cells^14^. Recent results allowed us to validate this method with a multidrug resistant clinical strain of *Enterobacter aerogenes* and an efflux pump deficient (Δ*tolC*) derivative, demonstrating the importance to use a robust internal control^17^.

The Innovative Medicine Initiative-funded consortium Translocation (www.imi.europa.eu/content/translocation) has defined as a main objective to study and dissect antibiotic translocation across bacterial membranes^11^. To achieve this goal, the consortium is setting new methods and new concepts to dissect this process^8^. Therefore, it is important to elaborate common standards and/or protocols for studying antibiotic accumulation and the correlation with the antibacterial activities in a series of well-defined isogenic strains. In this study, we propose a protocol for microspectrofluorimetry analyses on individual bacterial cells associated to biological/biochemical characterizations of appropriate whole bacterial samples. From this work, a new perspective emerges on how to capture and correlate the kinetics of antibiotic uptake inside bacterial cells in order to (i) understand the role of drug structure in the accumulation rate, and (ii) define the efficacy of the AcrB pump in relation to different quinolone structures during the time-course accumulation. This study contributes to the determination of parameters associated with the recently proposed concepts, “Resident Time Concentration Close to Target” (RTC2T) and “Structure Intracellular Concentration Activity Relationship” (SICAR)^8^.

## Results

### Antibiotic molecules

**Figure 1:**
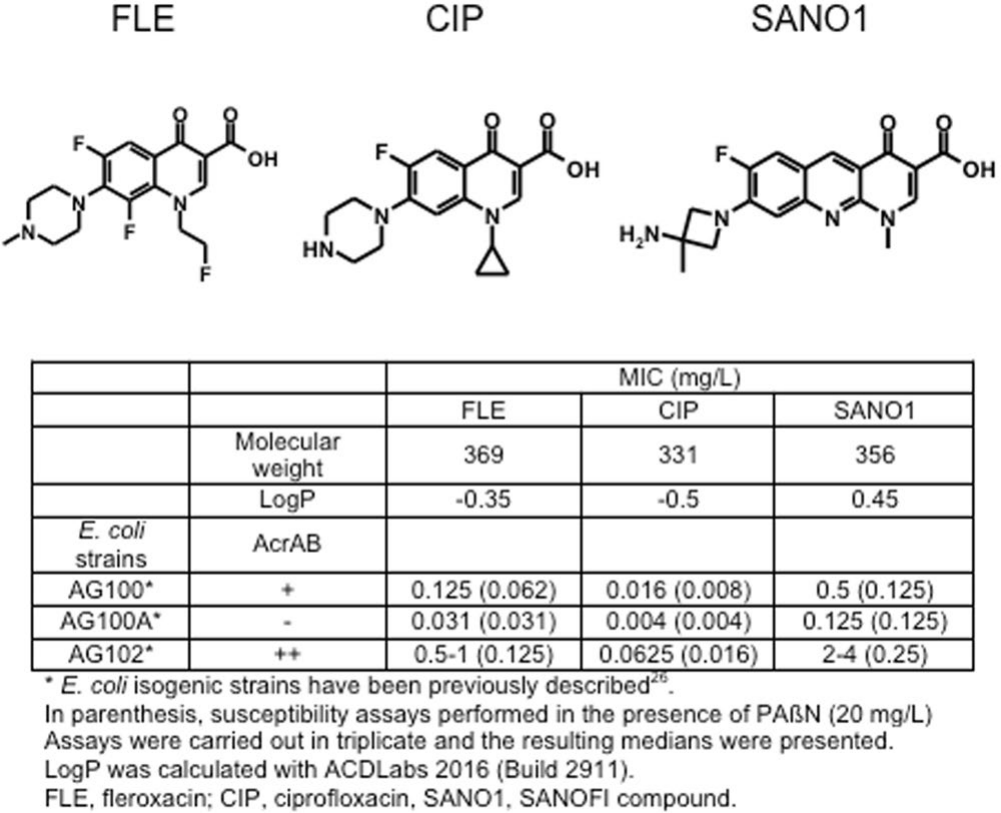
Chemical structure and antibacterial activities of fluoroquinolones used. SANO1 structure has been previously described^25^.

The structures of the three antibacterial molecules are presented in Fig. 1.

### Antibacterial susceptibility and AcrAB

The activity of FLE, CIP and SANO1 were assayed on the *E*. *coli* AG100 and AG100A (AcrAB- derivative) strains and AG102 strain that overproduces AcrAB pump (Fig. 1). In general, the susceptibility was higher with AG100A. In contrast, AG102 was more resistant. In the presence of PAßN, an efflux pump blocker used at low concentration^15^, the susceptibilities were noticeably increased whatever the fluoroquinolone assayed (Fig. 1). This suggests the involvement of AcrAB in the susceptibility level while in contrast, no increase of bacterial susceptibility was observed in the presence of membrane permeabilizer suggesting that the outer membrane was not a significant barrier in these strains (data not shown).

### Accumulation is dependent of AcrAB expression and of the fluoroquinolone

**Figure 2:**
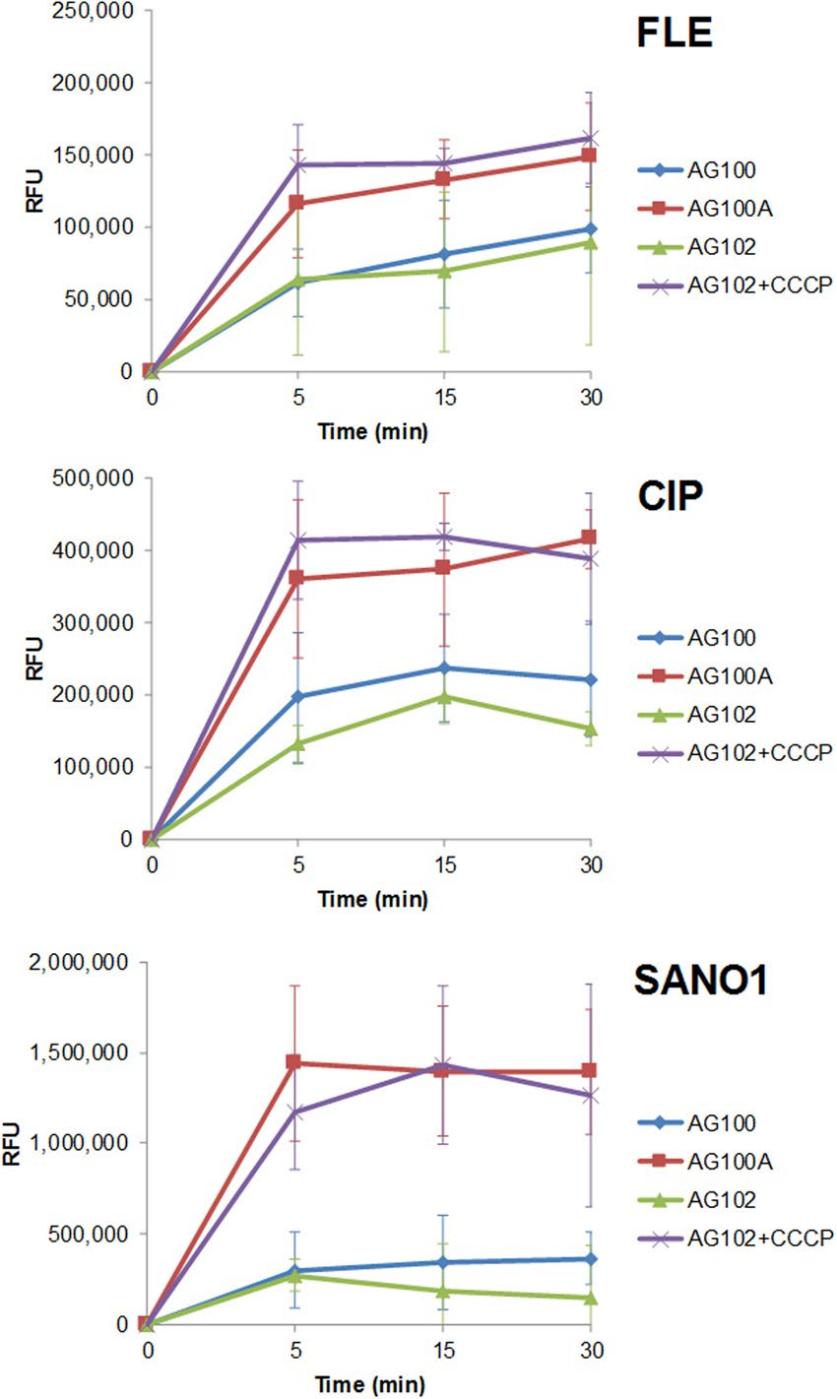
Intracellular concentration of Fleroxacin (FLE), Ciprofloxacin (CIP) and a benzo[b]napthyridone (SANO1) obtained with normalization by tryptophan. The accumulation was performed in AG100 (WT), AG100A (AcrAB−) and AG102 (AcrAB++) *E*. *coli* strains incubated 5, 15 and 30 min with 2 mg/L of molecules, in the absence or in the presence of CCCP (10 µM). The cells were lysated by HCl-glycine and the fluorescence signal (RFU) was plotted.

To correlate the antibacterial activity with drug uptake, the accumulations of the three molecules were studied in the three different strains. Figure 2 presented the results regarding the accumulation of FLE, CIP and SANO1 in bacterial population and demonstrated the effect of efflux expression on the intra-bacterial accumulation of the antibiotic: a very low content of the different drugs was observed in the AcrAB overproducer strain (AG102). When the incubation was performed in the presence of CCCP that collapses the energy driving force of efflux pump^8^, a significant increase of the accumulation was obtained in AG102 (Fig. 2). The plateau intensity reached level similar to the one we observed in the AcrAB deleted strain (AG100A). Moreover, in the parental strain, AG100, the fluorescence was less intense that the one obtained in AG100A suggesting that the basal efflux level present in this strain was sufficient to alter the accumulation inside the bacterial cell (Fig. 2 and Figure S1). It is important to note that the accumulation measured at 5 min in AcrAB- strain is related to the penetration capability for each tested molecule during early times of incubation in the absence of AcrAB pump activity. In contrast, the ratio of accumulation calculated at 15 min, AcrAB−/AcrAB++ or AcrAB-/AcrAB wild type, gives an illustration of the efflux capability to recognize the tested fluoroquinolones at steady state times of incubation^16^.

From the collected data (Figure S1 and Table 1), we can perform the following ranking:

- For 5 min incubation: in AcrAB- strain, SANO1 > CIP > FLE; in WT strain: SANO1 > CIP > FLE; in AcrAB overproducer strain: SANO1 > CIP > FLE.
- For 15 min incubation: in AcrAB- strain, SANO1 > CIP > FLE; in WT strain: SANO1 > CIP > FLE; in AcrAB overproducer strain: SANO1 > CIP > FLE.

**Table 1:** Accumulation of fluoroquinolones: SICAR^IN^ and SICAR^EF^ indexes.

Compound	SICAR^IN^			SICAR^EF^	
	Accumulated drug (10^−11^ µg/bacterial cell)			R	R′
	AG100A*	AG100*	AG102*	AG100A/AG100	AG100A/AG102
**5 min accumulation**					
FLE	0.80 (±0.25)	0.42 (±0.16)	0.44 (±0.07)	1.9	1.8
CIP	1.50 (±0.46)	0.82 (±0.37)	0.69 (±0.26)	1.8	2.2
SANO1	9.26 (±2.81)	2.5 (±1.62)	1.86 (±0.58)	3.7	5.0
**15 min accumulation**					
FLE	0.92 (±0.19)	0.56 (±0.26)	0.48 (±0.09)	1.6	1.9
CIP	1.55 (±0.46)	0.98 (±0.32)	0.83 (±0.12)	1.6	1.9
SANO1	9.12 (±2.41)	2.98 (±1.90)	1.27 (±0.008)	3.1	7.2

Intracellular accumulation of fluoroquinolones (FLE, CIP and SANO1, in 10^−11^ µg/bacterial cell). The accumulation was performed in AG100 (WT), AG100A (AcrAB-) and AG102 (AcrAB++) *E*. *coli* strains incubated 5 and 15 min with 2 mg/L of molecules in the absence and in the presence of CCCP (10 µM) and lysated by HCl-glycine. (Histograms are presented in Figure S1).

*Means of three independent assays performed in triplicates.

﻿﻿﻿﻿﻿﻿﻿﻿

The various values representing the ratio of fluoroquinolone accumulation, AG100A/AG100 and AG100A/AG102, R and R′ respectively, are presented in Table 1. These ratios give an evaluation of the efflux effect on the accumulation of the three fluoroquinolones.

Interestingly, the SANO1 molecule seemed to be very efficiently internalized at 5 min of incubation, 6–10 fold compared to CIP and FLE respectively, in the AcrAB- strain. However, it is also rapidly expelled by AcrB pump compared to FLE and CIP (Table 1) when we compared the accumulation ratio R or R′. This suggests that the molecule can have a better affinity for the AcrB sites and represents a better substrate for this pump. Importantly, no significant change in cell viability was observed during this period that corresponds to accumulation assay (Figure S3).

### Accumulation in individual bacterial cells

**Figure 3:**
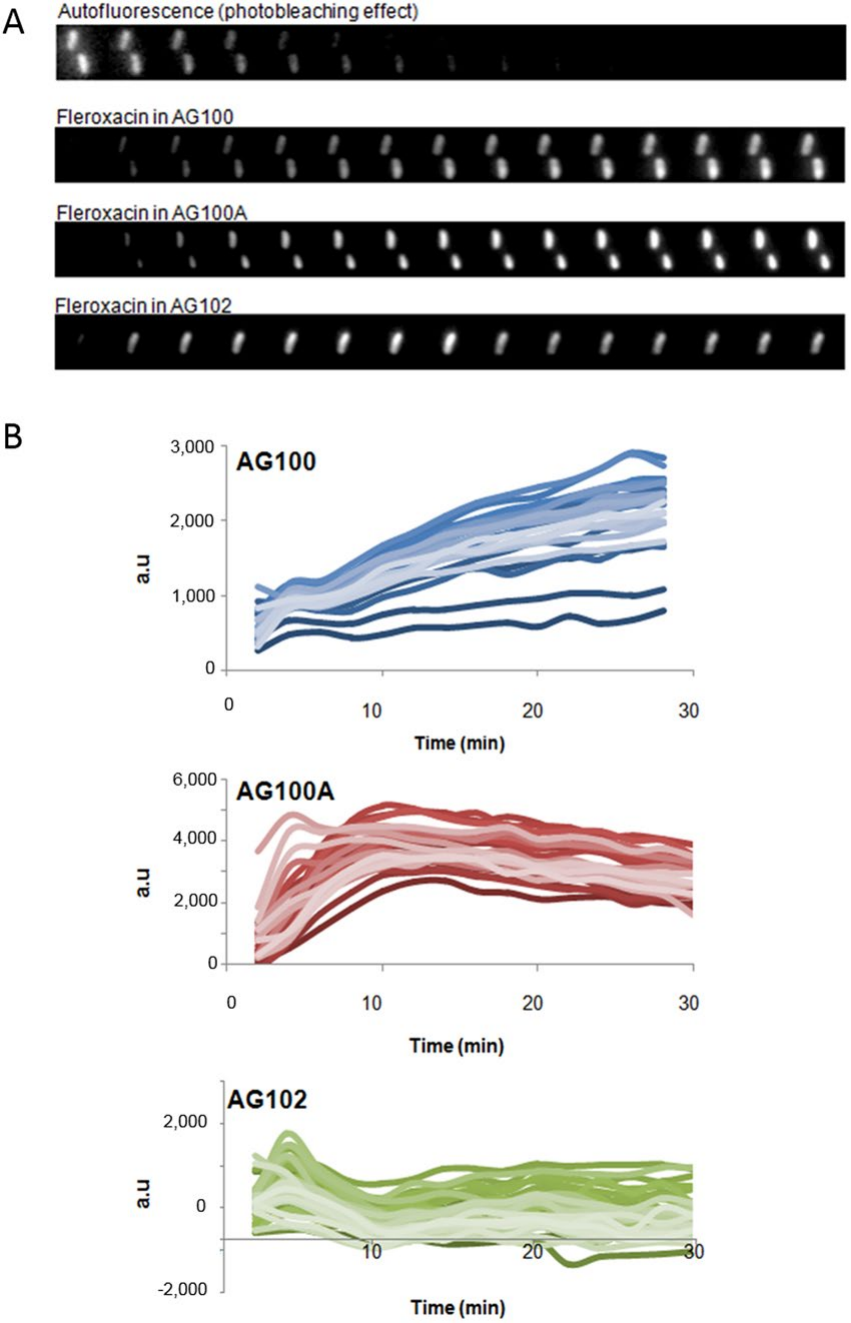
Microspectrofluorimetry on bacterial strains (30–40 bacterial cells) during time-course incubation. (**A**) Kinetics of FLE accumulation (2 mg/L) were followed in AG100, AG100A, AG102 isolated cell with a 2 min sampling time. (**B**) Kinetics of SANO1 accumulation in individual bacterial cells with 2 mg/L.

In order to follow the intracellular accumulation on individual bacterial cells, we performed time-lapse experiments on the DUV microscope. Bacteria were plated and observed for 30 min with a sampling time of 2 min: thumbnails of a bacterium incubated with FLE were plotted in Fig. 3A. Illustrations of CIP and SANO1 accumulation in individual bacterial cells under the same conditions were presented in Figure S4.

Fluorescence intensities corrected from background, photobleaching and crosstalking^17^ corresponding to about 30–40 individual cell measurements are presented in Fig. 3B. Interestingly, the steady state level of molecule (SANO1) accumulation in AcrAB- strain was obtained at 5–10 min under these conditions while a slow increase was observed in the parental strain expressing AcrAB basal level. In contrast, no significant accumulation was obtained in the AcrAB overproducer.

**Figure 4:**
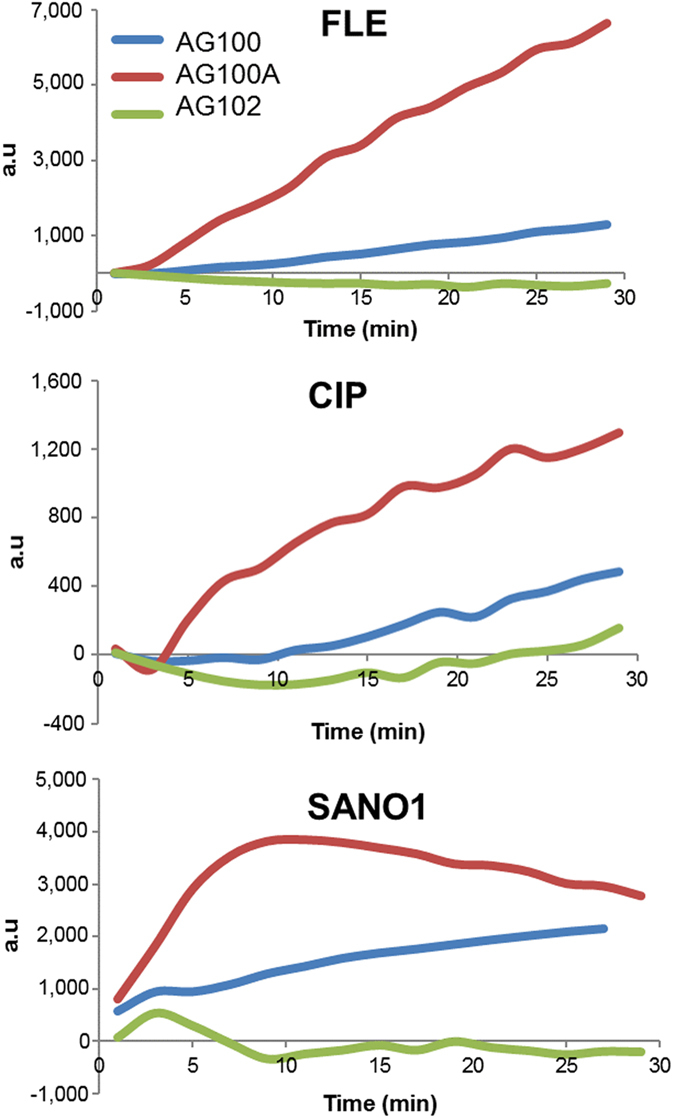
Microspectrofluorimetry on individual bacterial strains incubated with FLE/CIP/SANO1 at 2 mg/L calculated from curves as presented in Fig. 3B for SANO1.

We extended the analysis to other quinolone molecules and the means are presented in Fig. 4. Generally, no significant accumulation was noted in the AcrAB overproducer, whatever the molecule tested during the 30 min assay. Regarding the accumulation in parental strain, we observed a small increase with the three molecules, similar for FLE and SANO1, and reduced with CIP. Importantly, in the AcrAB deleted strain, the steady state was rapidly obtained for SANO1, after 10 min of incubation, whereas a continuous increase was noted with FLE and CIP during the same time-course accumulation assay (Fig. 4).

Regarding the profile of SANO1 accumulation, it is important to determine if the plateau observed at 10 min and followed by a curve reflecting a decrease accumulation in individual cells indicated a saturation process or a degradation of SANO1 inside the bacterial cell under these incubation conditions. To evaluate the stability of the compounds inside the cell, the molecules, FLE or SANO1, were incubated with a total bacterial lysate in the same conditions and the signal was monitored during 15 min. Interestingly, the signal recorded with FLE was stable during this interval when SANO1 exhibited an important decrease of the signal intensity during the incubation (Figure S5). This suggests that internalized SANO1 was less stable than FLE due to UV irradiation combined to the bacterial degradative activities or quenched during the binding to the target under these conditions.

## Discussion

With the continuous emergence of antibiotic resistance in Gram-negative bacteria, a main objective is to understand the relationships between antibiotic activity and the expression levels of efflux pumps expelling the penetrating molecules^7, 18–21^. A prominent question is therefore to relate the intra-bacterial accumulation and the chemical structure of the antibiotic^8^.

A variety of methods has been previously reported to follow the intracellular accumulation of antibiotics in *Enterobacteriaceae* expressing membrane-associated mechanisms of resistance including outer membrane impermeability or efflux pumps that expel all antibiotic classes^8, 12–15, 22, 23^. Until recently, no robust assay has been developed to study accumulation in individual bacterial cells. Cinquin *et al*.^17^ reported a fluorimetric method that allows the monitoring of the accumulation of fleroxacin in individual resistant bacteria^17^. This new methodology offers us the opportunity to examine in depth the time course accumulation by using a robust internal control (bacterial fluorescence) to standardize the signals and simultaneously correlate the imaging studies obtained with several strains to the variation of efflux activity in these strains. Moreover, this approach is not dependent to time-consuming protocols that can induce experimental artefacts^8^. It is important to adapt and use this original method to compare the accumulation of different quinolones in various efflux backgrounds and to try and correlate drug concentration and activity.

In this study, we used three molecules belonging to the structural group of fluoroquinolones and exhibiting different antibacterial activities against *E*. *coli* strains expressing various level of the AcrAB pump. Our aims were, (i) to determine the intra-bacterial concentration of ciprofloxacin, fleroxacin and an unmarketed fluoroquinolone of the benzo-napthyridone subfamily, SANO1, by conjointly using fluorimetric detection on bacterial population and individual bacterial cells; and (ii) to analyze the early steps of drug accumulation and the role of the AcrAB efflux pump. First of all, the values clearly indicate a MIC modulation strongly associated with the level of AcrAB in this collection: the susceptibility being comprised between 0.004 to 0.125 mg/L for the AcrAB- strain and to 0.0625 to 2 mg/L for the AcrAB overproducer, depending of the fluoroquinolone assayed. In parallel, we observe a marked difference in the accumulation profile depending on the quinolone structure. During the very early times of accumulation in individual bacterial cells devoid of efflux, SANO1 exhibits the highest rate of uptake compared to FLE and CIP, suggesting a well-adapted structure for permeation and a positive involvement of side chains in membrane translocation.

However, SANO1 is also very well recognized by the AcrB pump, since it is rapidly and efficiently expelled as reported by the ratio of signals observed in efflux producer strain in the absence and in the presence of CCCP, an ionophore that collapses the driving force of AcrAB-TolC^9, 10^. Regarding CIP and FLE, the accumulation is also impaired in the AcrAB overproducer strain compared to AcrAB- strain, but to a lesser extent compared to SANO1. Regarding the curves, it is important to mention that we observed an effect on accumulation rate for the first incubation time (compare AG100A to AG100 curve) suggesting that the efflux activity is observed early. This suggests that, as soon as drug molecules permeate the cell envelope, AcrB transporter recognizes them and pumps antibiotics out of the cell. This is the case for the parental strain and for the overproducer strain with a noticeable shift in the curve at longer incubation times for the latter that reflects an additional reduction in the accumulation rate. These observations, also noted in individual cell assays, suggest that the activity of AcrB pump could be partly saturated when expressed at a basal level. This efflux impact on the molecule accumulation is correlated to the antibiotic susceptibility determined in these strains, although the MIC are recorded after 18 hours of incubation, a period that could buffered the effect of different accumulation rates. In addition, the effect of PAßN, which has been reported as a pump blocker at low concentration^10, 24^, on the antibiotic susceptibility is in accordance with accumulation studies carried out with these strains expressing various level of AcrAB efflux transporter.

It is tempting to correlate the accumulation ratio obtained in each strain with their susceptibility, but it is important to mention that the time scale is different, few minutes compared to an overnight incubation. Moreover, we have selected a ratio fluoroquinolone concentration/bacterial number that is different to the recommendation rules used for MIC determination in order to have a limited effect on cell viability during the experimentation times. One objective of IMI-Translocation consortium is to develop an assay allowing us to simultaneously measure antibiotic concentration inside the bacterial cells and rate-killing^8, 11^.

This is the first time that we studied the real time accumulation of antibiotics belonging to the same structural family in various efflux pump backgrounds. If we calculate the impact of efflux level on the accumulation of the three fluoroquinolones, from efflux minus to overexpressed efflux background, called R and R′ respectively in Table 1, we obtain a specific index that reflects the relationships between the chemical structure of the molecule and the efflux activity on the accumulation level. This index fits in well with the new concept “Structure Intracellular Concentration Activity Relationship” (SICAR) recently proposed^8^. Practically, the SICAR connects the physicochemical drug properties to the efficacy of translocation through the bacterial membrane and the resulting intracellular accumulation. With this study, we can conclude that: (i) SANO1 exhibits a high penetration rate (9.26 10^−11^ µg/bacterial cell at 5 min) corresponding to a favorable SICAR influx index (SICAR^IN^ defined as the intracellular concentration obtained in the AcrAB- strain during 5 min of incubation), better than the corresponding index of CIP and FLE; and (ii), in contrast, the CIP and FLE present a quasi-similar SICAR efflux index (SICAR^EF^ defined as the ratio of molecule accumulated in AcrAB- strain to AcrAB producer strain) while SANO1 exhibits an unfavorable SICAR^EF^ that reflects a high level of sensitivity to efflux transporter (AcrB in this case). In the case of SANO1, several exposed chemical side chains could be involved in the affinity for AcrB binding sites when we compare the three structures. These SICAR indexes can be used to precise the chemotypes that are related to permeation of different antibiotics belonging to the same group. These indexes could be determined for other antibiotic classes by extending the assay to mass spectrometry analyses in order to take into account non-fluorescent molecules^14^.

A further study could be to sequentially modulate these putative pharmacophoric groups in order to precise their respective involvement in influx versus efflux, and to integrate the affinity constant in the matrix to complete the SICAR index. Conjointly to the use of adjuvants^26–30^ in combination to restore antibiotic susceptibility, this study opens a way to circumvent the role of membrane permeability and efflux transporters in resistance by a rationale pharmacomodulation taking into account the molecular profile, SICAR index, of antibiotic molecules.

## Methods

### Bacteria and Media

*Escherichia coli* strains used in this study are listed in Fig. 1.

### Drug susceptibility assays

Fleroxacin (FLE), ciprofloxacin (CIP) and benzoquinolone (SANO1) were assayed to study the antibiotic susceptibility of *E*. *coli* strains. MIC values of antibiotics were determined by the microdilution method (CLSI) in liquid Mueller Hinton II media by using the twofold standard microbroth dilution method (microplates and automatic analyses Tecan®) (CLSI, http://clsi.org/). When required the MICs were determined in the presence of the phenylalanine arginine β -naphthylamide (PAβN) used at 20 mg/L. MIC values were read after 18 h of incubation at 37 °C. Experiments were carried out in triplicate and the resulting medians were presented.

### Accumulation protocol

Bacteria grown at 37 °C in Luria-Bertani broth to mid-exponential phase (corresponding to 0.6 optical density units at 600 nm) were concentrated 10-fold. Briefly, the bacterial suspension was centrifuged at 6,000 × *g* for 15 min at 20 °C and pellets were re-suspended in 1/10 of the initial volume in sodium phosphate buffer (50 mM) at pH 7 supplemented with MgCl_2_ (5 mM) (NaPi-MgCl_2_ buffer) to obtain a density of 6 10^9^ CFU.ml^−1^. In culture tubes, 4 ml of the bacterial suspension was incubated 5, 15 and 30 min at 37 °C (final volume 5 ml) with FLE, CIP or SANO1 at 2 mg/L, in the absence or in the presence of the efflux blocker CCCP used at 10 μM that collapses the energy-driven force needed by the efflux pump^15^. Bacterial suspensions incubated without antibiotics, with or without CCCP, were used as controls. Suspensions (800 μL) were then loaded on 1 M sucrose cushion (1,100 μL) and centrifuged at 9,000 × *g* for 5 min at 4 °C to eliminate extracellular-adsorbed compounds and to collect the washed bacteria.

After centrifugation, pellets corresponding to 800 μL of bacterial suspensions were lysed with 500 μL of 0.1 M Glycin-HCl pH 3 overnight at room temperature. After a centrifugation for 15 min at 9,000 × *g* at 4 °C, 400 μL of lysates were mixed with 600 μL of Glycin-HCl buffer (FLE and CIP) or 3 M Tris pH 8.8 buffer (SANO1) and analyzed by spectrofluorimetry (Ex 290 nm, Em 420–480 for FLE; Ex 275 nm, Em 420–480 nm for CIP; Ex 275 nm, Em 499–529 for SANO1). Calibration curves were carried out to determine the quantity of molecules accumulated per cell. Various concentrations of FLE, CIP or SANO1 were mixed with bacteria lysates at OD = 4.8 and measured with spectrofluorimeter (n = 3).

To control that bacterial cells are alive during the experimental time, the number of colony forming units (CFU) were determined by sampling the bacterial suspension during antibiotic incubation. Small volumes were collected and dilutions were performed and plated on Petri dishes. CFUs were measured and survival ratios, with and without fluoroquinolone, were generated. The assays were performed in triplicate. No significant change in cell viability was observed during this period that corresponds to accumulation assay.

It must be noted that the ratio cell/antibiotic concentration were different in the MIC assay and in accumulation assay carried out using starving conditions during a limited incubation time (5–30 min) as previously determined^17^.

### DUV Fluorescence accumulation in individual bacterial cells

The accumulation in individual bacterial cells was monitored directly under the deep ultraviolet (DUV) microscope during 30 min. Bacteria were concentrated in order to obtain an OD of 4.8 in NaPi-MgCl_2_ buffer. Then 120 µL of the suspension were centrifuged on a 1 M sucrose cushion (165 μL) at 9,000 × *g* for 5 min at 4 °C. The pellets were resuspended extemporaneously in 40 µL of NaPi-MgCl_2_ buffer containing or not FLE, CIP or SANO1 (2 µg/mL) in the absence or in the presence of CCCP (10 µM). 0.5 µL of resuspended pellets were deposited between two quartz coverslips and analyzed by DUV fluorescence imaging at DISCO Beamline. Bacterial cells were first located in brightfield before excitation in DUV under a microscope (Zeiss Axio Observer Z-1) at Synchrotron SOLEIL^31^. Emission was collected through a Zeiss ultrafluar objective at 100x with glycerin immersion. The FLE/CIP/SANO1 fluorescence was recorded by exciting at 290/275/275 nm, using a dichroic mirror at 300 nm (OMEGA Optical, Inc., USA) through an emission bandpass filter at 420–480 nm (OMEGA Optical, Inc., USA) for FLE and CIP and a 499–529 nm (SEMROCK) bandpass filter for SANO1. For the tryptophan fluorescence, the emission was passing through an emission bandpass filter at 327–353 nm (SEMROCK). Fluorescence images were recorded by a BUV EM CCD (Princeton PIXIS 1024 BUV). The whole setup (microscope, stages, filters, camera) was controlled by Micro-Manager^32^. Bacteria were observed for 30 min with a sampling time of 2 min for each area: 30 s with the filter 1 (fluoroquinolone molecule), 30 s with the filter 2 (tryptophan), followed by 60 s of pause. During the intervening pause in sampling, the same acquisition cycle was performed on another bacterium, avoiding constant UV irradiation of the same field.

The images were analyzed with Image J (Rasband, W.S., ImageJ, U. S. National Institutes of Health, Bethesda, Maryland, USA, http://imagej.nih.gov/ij/)^33^. The illumination heterogeneities were corrected before background subtraction. First, threshold was automatically adjusted using a triangle algorithm; thereafter, bacteria were analyzed as the remaining particles. The mean intensity coming from each bacterium was automatically calculated considering its pixel area. Finally, all bacteria signal taken from one image were averaged. For each condition, two different localizations with minimum 30 bacteria per field of view were recorded and averaged.

### Stability assays

UV irradiation and incubation inside bacteria can promote some degradation of chemicals. In order to check the stability of fluoroquinolone during the assays, incubations of FLE and SANO1 were performed using a bacterial lysate obtained after cell disruption of AG100A strain using the method previously described^15^. The same conditions of incubation (lysate corresponding to same cell density, drug concentration, UV irradiation exposure) were used and the fluorescence was recorded each min during the assay. The percentage of fluoroquinolone stability was calculated using the initial fluorescence obtained at 0 min incubation as 100% for each molecule.

## Electronic supplementary material

Supplementary Information
