# Supplementary material for: Fluoroquinolone structure and translocation flux across bacterial membrane
Source: Sci Rep. 2017 Aug 29;7:9821. doi: 10.1038/s41598-017-08775-4 (PMC5575017; doi:10.1038/s41598-017-08775-4)

SUPPLEMENTARY INFORMATION

Fluoroquinolone structure and translocation flux across bacterial membrane

Julia Vergalli[a,*], Estelle Dumont[a,*], Bertrand Cinquin[b], Laure Maigre[a], Jelena Pajovic[b], Eric Bacqué[c], Michael Mourez[c], Matthieu Réfregiers[b], Jean-Marie Pagès[a,*]

Figure S1: Measure of accumulation in bacterial population
Intracellular concentration of Fleroxacin (FLE), Ciprofloxacin (CIP) and Sanofi1 (SANO1) obtained with normalization by tryptophan and by the slope of their respective standard curve (Figure S2). The accumulation was performed in AG100 (WT), AG100A (AcrAB-) and AG102 (AcrAB++) *E. coli* strains incubated 5, 15 and 30 min with 2 mg/L of molecules +/- CCCP (10 µM) and lysated by HCl-glycine.

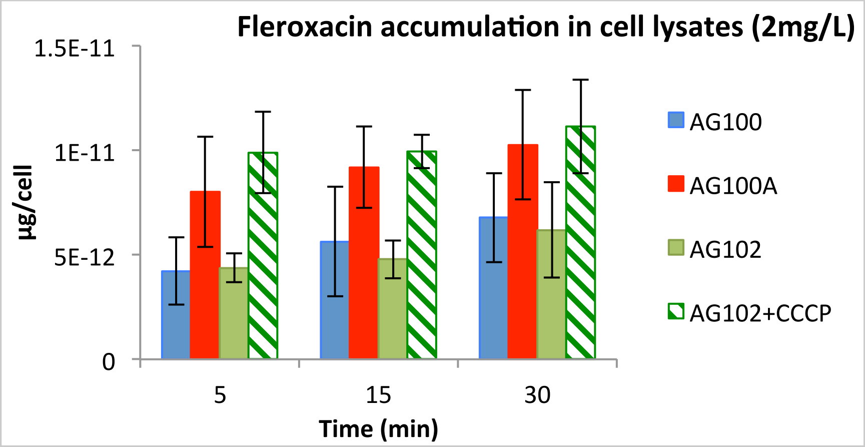

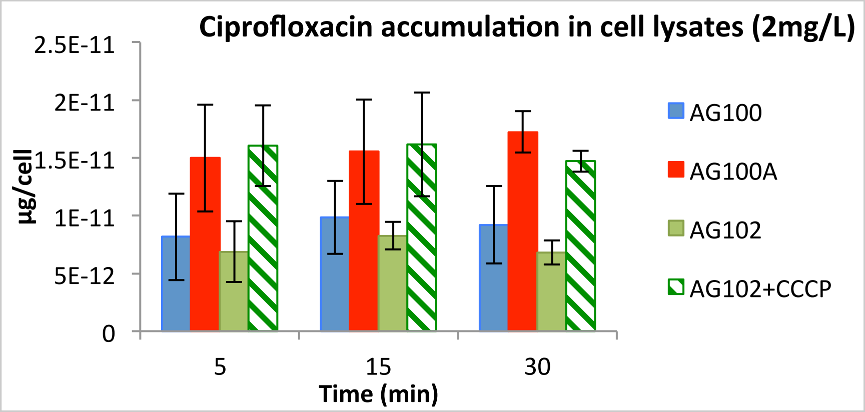

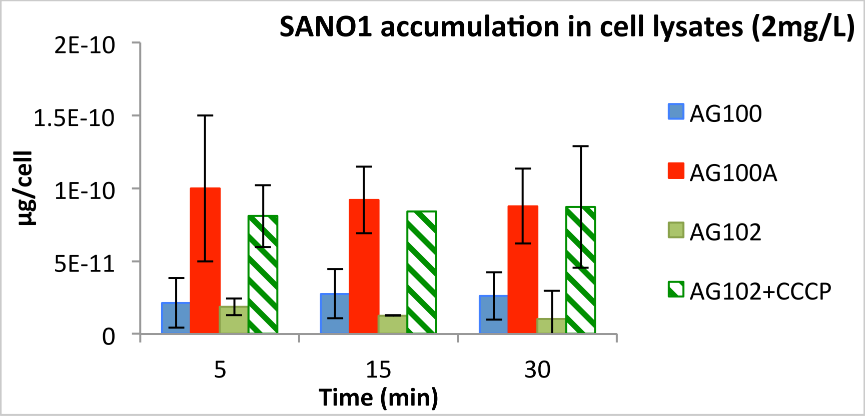

Figure S2: Standard calibration curves for FLE, CIP and SANO1 fluorescence intensity.

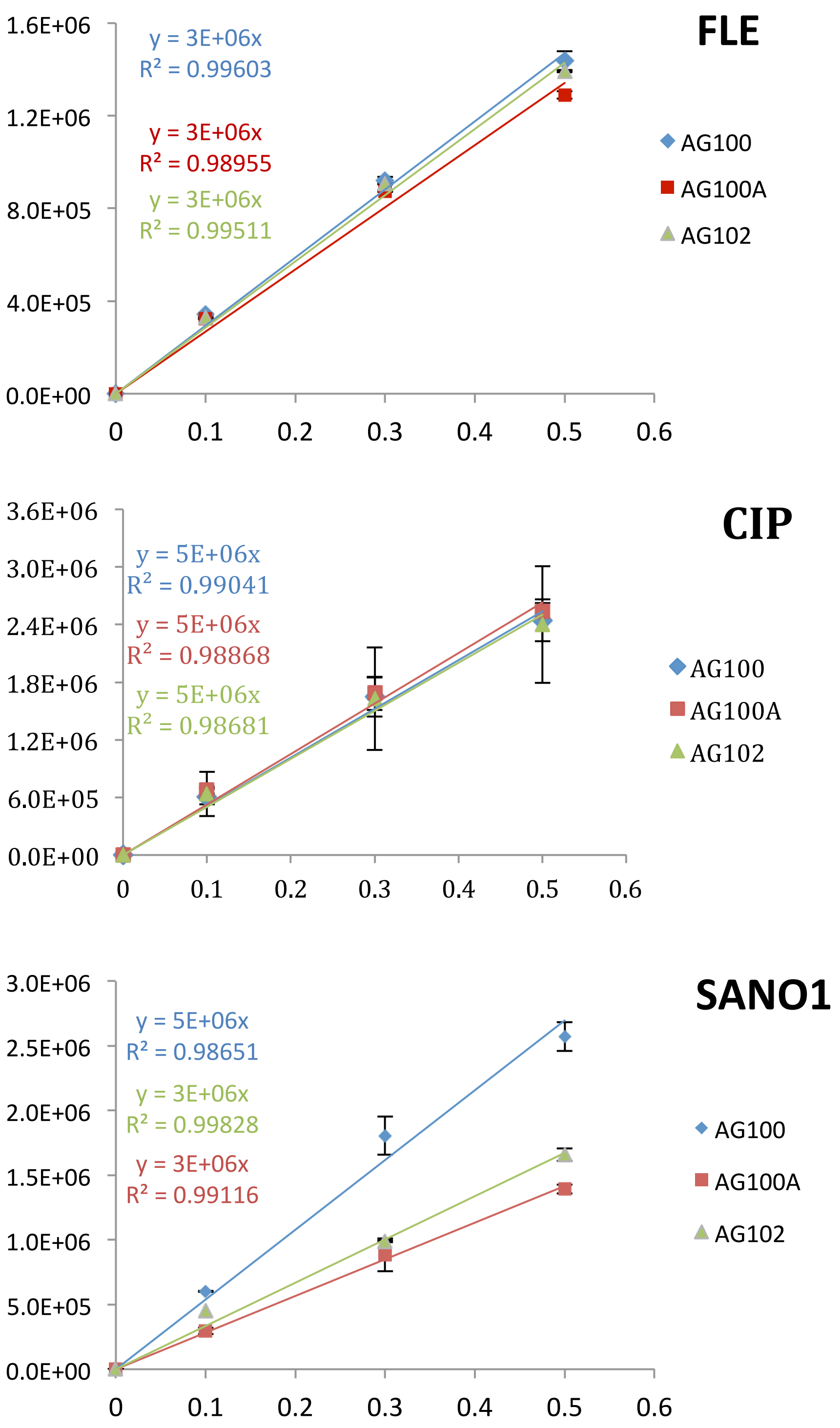

Figure S3: Bacterial survival in AG100 series during antibibiotic accumulation.

The percentage (%) of bacterial survival (in AG100, AG100A and AG102) was monitored after incubation with 2 mg/L of FLE for 30 min. Assays were carried out in triplicate and the resulting means were presented.

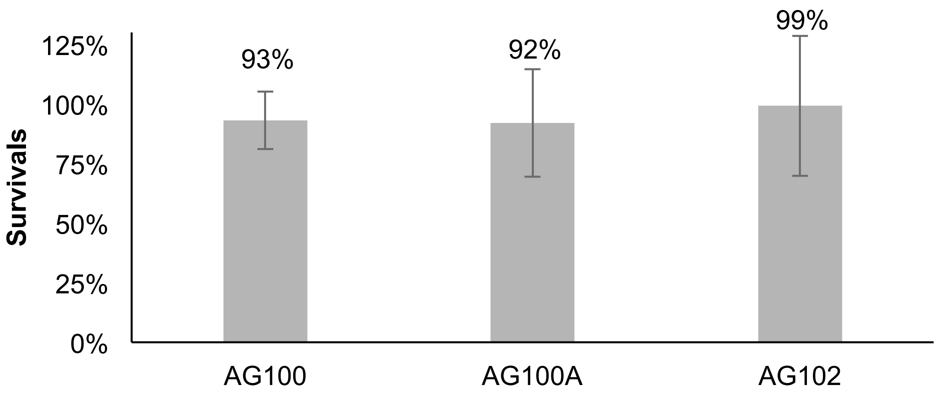

Figure S4: Kinetics of CIP (**A**) and SANO1 (**B**) accumulation (2 mg/L) in AG100, AG100A, AG102 isolated cells, measured with microspectrofluorimetry

A

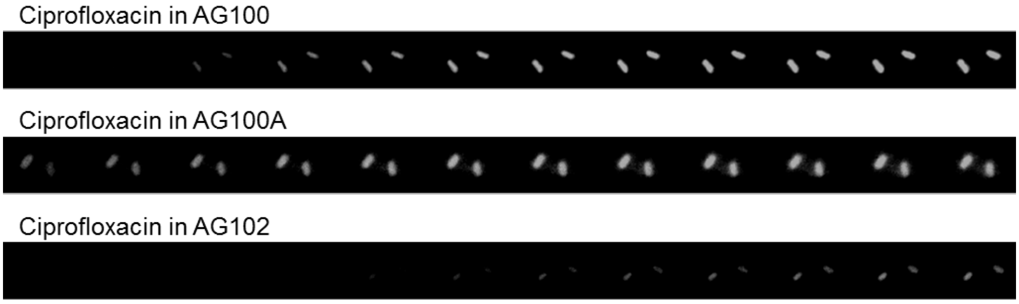

B

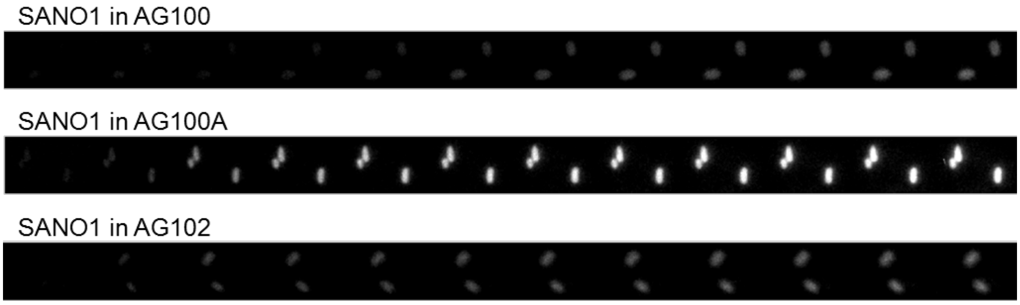

Figure S5: Stability of FLE and SANO1
Lysate suspension of AG100A was incubated with FLE or SANO1 and treated under the same conditions used in Figure 4. The fluorescence intensity was recorded each minute during the incubation and plotted as percentage of the initial value measured at t=0 for each molecule.

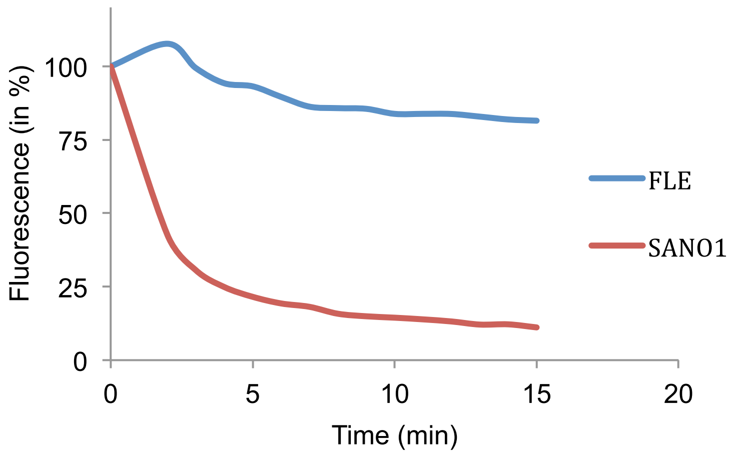

Supplement: Supplementary file 1 — Supplementary Information [file 41598_2017_8775_MOESM1_ESM.pdf]
